# Role and plasticity of Th1 and Th17 responses in immunity to *Staphylococcus aureus*

**DOI:** 10.1080/21645515.2019.1613126

**Published:** 2019-10-31

**Authors:** Alessandra Ferraro, Sofia M. Buonocore, Philippe Auquier, Isabelle Nicolas, Hugues Wallemacq, Dominique Boutriau, Robbert G. van der Most

**Affiliations:** GSK Vaccines, GSK, Rixensart, Belgium

**Keywords:** CD4^+^ T cells, Th17, anti-bacterial immunity, commensal, vaccine, plasticity

## Abstract

The human commensal *Staphylococcus aureus* (SA) is a leading cause of skin/soft tissue and surgical-site infections, and bacteremia. Functional antibodies and T-cell-mediated immunity, particularly Th1/Th17 responses, are thought to mediate protection. Vaccine development may be hindered by modulation of vaccine-induced T cells by pathogen-activated immunoregulatory responses, e.g., via IL-10.

We screened SA proteins for CD4^+^ T-cell-activating and IL-10/IL-17-inducing capacities using healthy donor-derived PBMCs. Responses were characterized (Th1/Th17/Th22/immunosuppressive IL-10-producing cells) using intracellular cytokine staining and flow cytometry. Phenotypic plasticity of Th1/Th17 cells was evaluated under pro- or anti-inflammatory conditions using modulatory cytokines. The impact of vaccination on SA-specific memory responses was assessed using samples from a clinical trial evaluating AS03-adjuvanted and non-adjuvanted multicomponent (CPS5/CPS8/α-toxin/ClfA) vaccines (NCT01160172).

The donors exhibited SA-specific memory T-cell responses, indicative of pre-existing immunity to SA. We identified effective activators of Th1 responses (EbhA/IsaA/SdrE/MntC/Aaa/α-toxin), and Th17 and Th1/Th17 responses (EbhA/IsaA/SdrE and, to a lesser extent, α-toxin), but not of Th22 responses or IL-10 production. MRPII, IsdA, and ClfA were inefficient CD4^+^ T-cell activators in our assays. IL-10, likely produced by innate immune cells, influenced mainly Th1 cells by suppressing IFN-γ production. The memory CD4^+^ T-cells observed after long-term stimulation with α-toxin and ClfA indicated that vaccination with these proteins had induced expansion of pre-existing Th1 but not Th17 responses, without apparent adjuvant effect, confirming the trial data. The Th1/Th17-driving proteins (EbhA/IsaA/SdrE) shared low IL-10-promoting abilities and restricted phenotypic plasticity under pro- and anti-inflammatory conditions.

Given the complex immunopathology and multiple virulence factors, identification of Th1/Th17-driving antigens, adjuvants and administration routes, and delineation of the role of memory responses, may advance vaccine development.

## Introduction

*Staphylococcus aureus* (SA) is a human commensal often carried on the skin and in the nose, but has a high pathogenic potential when present in skin lesions or in the bloodstream. It is a leading cause of skin and soft tissue infections (SSTI), surgical-site infections and bacteremia. SA causes serious disease burden in community settings, and acts as a nosocomial pathogen in health-care settings. No immune mechanism of protection has been defined. It is thought that both functional antibodies (opsonizing bacteria or neutralizing virulence factors) and T cell-mediated immunity would constitute an efficacious adaptive immune response, with a contributing role for innate immunity including immunological memory developed by innate immune cells.^1^^–3^ While the optimal relative contributions of these responses to protection have not been delineated for humans, murine and human data suggest that CD4^+^ T cells are particularly critical when antibody responses are low.^4–6^ Healthy individuals can exhibit memory responses targeting several SA antigens, which may influence the course of bacteremia.^7–9^

Mouse models have been shown to be inadequate to accurately predict the success of human SA vaccine candidates, and to date, none of these candidates have demonstrated efficacy in humans.^2,3,10^ Indeed, vaccines designed to induce functional antibodies targeting the virulence factors capsular polysaccharide types 5 and 8 (CPS5 and CPS8^11^), or iron-regulated surface protein B (IsdB; an SA extracellular protein involved in iron acquisition^12^), failed to show consistent protection.^13–15^ Vaccines that are or were in Phase II trials include an SA adhesin homolog derived from *Candida albicans* protein Als3p,^16^ and a multiple-component vaccine containing CPS5 and CPS8 glycoconjugates combined with clumping factor A (ClfA) and MntC.^17^ These vaccines elicited antibody responses, but, with the exception of Als3p, no substantial antigen-specific T-cell responses.^16,17^ Several other candidate vaccines are in preclinical or Phase I development stages (reviewed in ref.^2,3^).

CD4^+^ T cells have a helper function for antibody responses, and cytokines produced by effector CD4^+^ T cells, such as interleukin (IL)-17A (hereafter referred to as IL-17), induce recruitment and activation of innate immune cells, which also have a role in protection.^1,18^ In mice, systemic T helper (Th) 1 responses have been associated with protection against bacteremia, and homing of Th17 cells to the skin-mediated protection against SSTI, while dysregulation of systemic IL-17 responses has been linked to pathological effects.^7,19–22^ The high susceptibility to SSTI of individuals with conditions resulting in deficient Th17 responses (e.g., HIV infection with low CD4^+^ T-cell counts, hyper-immunoglobulin E [‘Job’s’] syndrome, or atopic dermatitis), suggests that Th17 cells also have a protective role against human SSTI.^23,24^ However, since Th1 and Th17 responses are usually induced concomitantly, their individual roles in protection are not fully distinguishable. Moreover, Th17 cells, which secrete IL-17, IL-17F and IL-22, can display phenotypic plasticity in response to SA and acquire an immunoregulatory phenotype.^25^

SA cell-wall components and secreted toxins can modulate the immune response to promote either disease tolerance or immune evasion.^8^ In response to SA, innate cells (particularly monocytes and macrophages) and T cells can produce the anti-inflammatory cytokine IL-10,^8,26^ which dampens pro-inflammatory cytokine responses and pathogen-specific Th1/Th17 responses.^27,28^ Correspondingly, high levels of circulating IL-10 and lack of the Th17-polarizing cytokine IL-1β have been linked to increased mortality in SA bacteremia patients.^29^

The complexity of SA-specific immunity implies that successful vaccine development benefits from a better understanding of the functional properties and plasticity of anti-bacterial CD4^+^ T cell lineages. Since these properties vary between bacterial proteins, and given the paucity of known SA T-cell antigens and the inadequacy of preclinical SA models of infection, we screened several proteins for their CD4^+^ T-cell-activating and IL-10-inducing capacities in human cells, by characterizing the response phenotypes (i.e., Th1, Th17 or Th22 lineages or immunosuppressive IL-10-producing CD4^+^ T cells) observed upon stimulation with these proteins. For antigens shown to induce Th1/Th17 responses, we then studied the plasticity of the response under pro- or anti-inflammatory conditions, to gain insight in how cytokines, particularly IL-10, modulate Th1 and Th17 responses. Finally, we assessed the impact of vaccination on the SA-specific memory pool, using samples from a Phase I study evaluating an investigational multicomponent vaccine combining tetanus toxoid (TT)-conjugated CPS5 and CPS8 with mutated detoxified α-toxin (AT) and ClfA.^30^

## Results

Unless specified otherwise, peripheral blood mononuclear cells (PBMCs) were derived from healthy donors (N = 14 to 16 per experiment) who were on average 49 years of age (range 26–67), predominantly (70%) male, and of unknown carriage status. SA-specific CD4^+^ T-cell responses and cytokine production in culture supernatants were measured using intracellular cytokine staining (ICS) and cytometric bead array (CBA), respectively. Baculovirus-expressed malaria thrombospondin-related anonymous protein (TRAP) was included as negative control, and TT and inactivated SA (killed whole-cell antigen; KWC) were used as positive controls.

### Screening of CD4^+^ T-cell antigens

**Figure 1.**
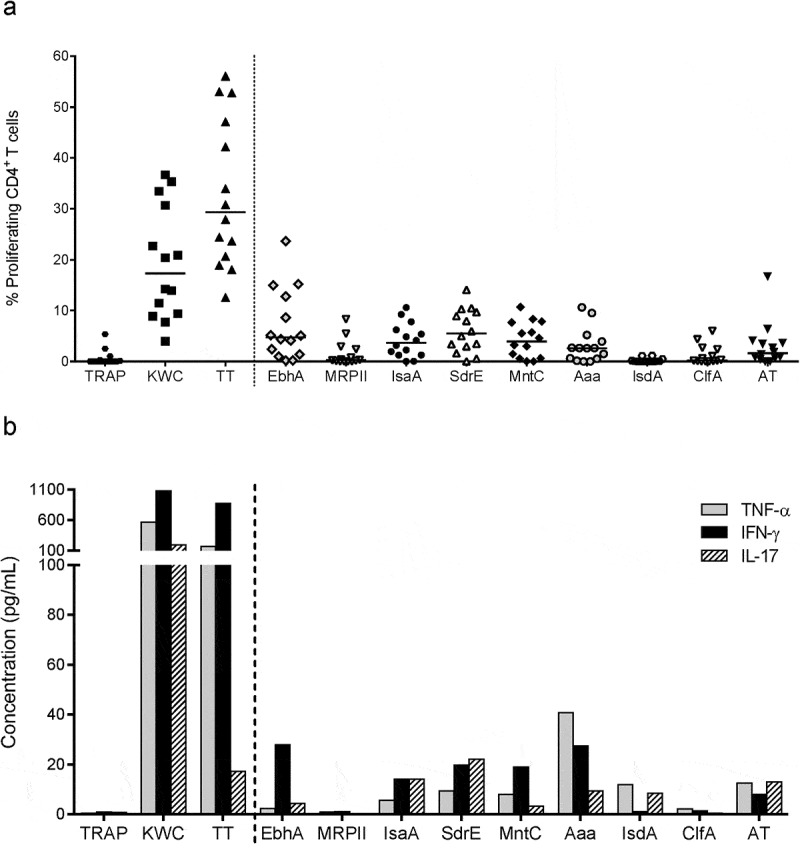
**CD4^+^ T-cell activation by SA proteins**. Background (medium)-subtracted SA-specific responses from healthy donor PBMCs cultured with controls or SA antigens are represented in terms of either the frequencies of proliferating cells in the CD4^+^ T cells (**a**), or the cytokine (IFN-γ, IL-17, TNF-α) production measured in culture supernatants (**b**). TT, tetanus toxoid (positive control). KWC, SA killed whole-cell antigen (positive control). TRAP, malaria thrombospondin-related anonymous protein (negative control). Each symbol in (**a**) represents one subject. Horizontal lines (**a**) and bars (**b**) represent medians calculated for the 14 to 16 donors included in each experiment.

Based on the presence of cell surface-expressed protein features, functional domains and levels of intra-species conservation, we selected nine proteins for production in *Escherichia coli*, purification, and evaluation (Table 1). Six antigens (EbhA, IsaA, SdrE, MntC, Aaa and AT) were shown to induce both specific CD4^+^ T-cell proliferation and secretion of at least one cytokine, among interferon (IFN)-γ, IL-17 and tumor necrosis factor (TNF)-α, in most of the donors (Figure 1(a,b)).

**Table 1.** Evaluated *Staphylococcus aureus* proteins.

				Conservation^b^	
Name	Abbreviation	Category	Functional class^a^	Range (%)	Occurrence
Extracellular matrix-binding protein homologue A	EbhA	SP	ECBP homologue A	90–100	7
Monovalent cation/proton antiporter (fmtB) II	MRPII	SP	Resistance protein	69–100	7
Immunodominant staphylococcal antigen A	IsaA	SP	unknown	99–100	7
Serine aspartate repeat protein E	SdrE	SP	ECBP; binds complement regulator factor H	85–100	6
Manganese ATP-binding cassette transporter C	MntC	SP	ECBP; Mn transport protein	99–100	7
Autolysin/adhesin	Aaa	SP	ECBP; binds fibronectin, vitronectin and fibrinogen	80–100	7
Cell wall protein Seg7	IsdA	SP	Iron capture; heme-binding protein	92–100	7
Clumping factor A	ClfA	SP	ECBP; fibrinogen-binding factor A	83–100	6
α-hemolysin H35R	AT	Toxin	Cytolytic pore-forming toxin	98–100	7

^a^Information based on refs.^31–36^  bIntra-species conservation of the nine Staphylococcus aureus (SA) antigens of strain ATCC35556/NCTC8325 was assessed in-house by translating their nucleotide sequences into amino acid sequences, and performing similarity analyses by multiple alignment comparisons using ClustalX software. The percentage identity between the sequences was defined as the ‘(number of identical residues/length of alignment) × 100’. ‘Occurrence’ denotes the number of SA strains (among the seven SA strains in the analysis) for which the sequence in question was found to be present, and which contributed to the associated percentage identity range. ECBP, extracellular component-binding protein. SP, surface protein.

### Characterization of specific CD4^+^ T-cell responses

**Figure 2.**
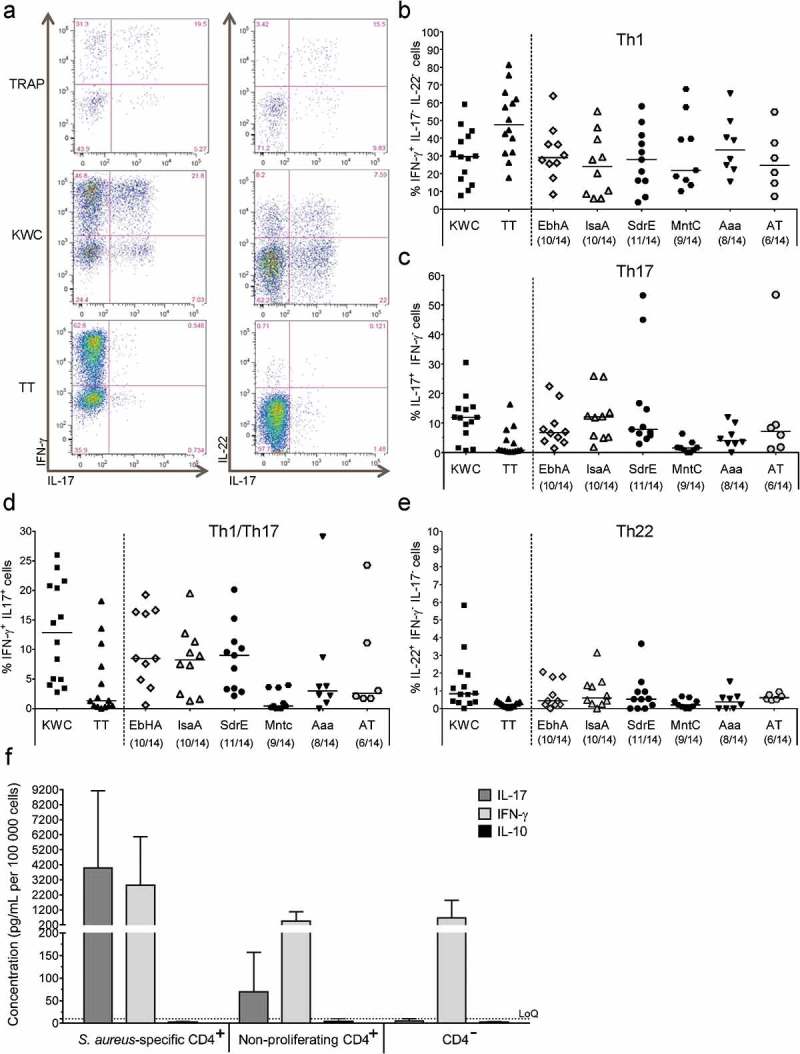
**Intracellular cytokine expression in SA-specific CD4^+^ T cells**. (**a**) The gating strategy to identify the specific cell-mediated immune responses by intracellular cytokine detection is presented. Flow cytometry was used upon 7 days of *in vitro* stimulation of PBMCs with the positive and negative controls. Gating was performed on CD4^+^CellTrace^low^ cells. The numbers in the quadrant gates of the plots denominate each distinct population based on their cytokine (IFN-γ, IL-17, and IL-22) production. The presented results are considered representative of the range of responses obtained for all subjects. TT, tetanus toxoid (positive control). KWC, SA killed whole-cell antigen (positive control). TRAP, malaria thrombospondin-related anonymous protein (negative control). (**b-e**) Background (medium)-subtracted SA-specific CD4^+^ T-cell responses from PBMCs from healthy donors (N = 14) in terms of the frequencies of cytokine-producing cells (i.e., IFN-γ^+^ IL-17^−^ IL-22^−^ [Th1], IL-17^+^ IFN-γ^−^ [Th17], IFN-γ^+^ IL-17^+^ [Th1/Th17] and IL-22^+^ IFN-γ^−^ IL-17^−^ [Th22] cells) in proliferating CD4^+^ T cells are presented. Each symbol represents one individual. Lines represent medians. (**f**) Median IL-17, IFN-γ, and IL-10 concentrations in culture supernatants of KWC-stimulated PBMCs are represented. The KWC-stimulated PBMCs were polyclonally activated, and sorted into populations of SA-specific proliferating CD4^+^ T cells, non–proliferating CD4^+^ T cells, and non-CD4^+^ (CD4^−^) cells. Results from three independent experiments performed for a total of six donors are shown. LoQ: Limit of Quantification.

Since the T-cell phenotype determines both the protective and pathologic qualities of the response, we evaluated for the six identified CD4^+^ T-cell antigens, their intrinsic capacity to activate cells with a pro-inflammatory (Th1, Th22 or Th17/Th1) or immunosuppressive (Th17/IL-10) profile. The gating strategy is presented in Figure 2a.

All antigens induced Th1 (IFN-γ^+^ IL-17^−^ IL-22^−^) responses in the majority of the donors. AT (albeit poorly), EbhA, IsaA, and SdrE induced Th17 (IL-17^+^ IFN-γ^−^) responses, and EbhA, IsaA, and SdrE also induced Th1/Th17 (IL-17^+^ IFN-γ^+^) responses, but none of the proteins induced consistent Th22 (IL-22^+^ IL-17^−^ IFN-γ^−^) responses (Figure 2(b–e)).

No IL-10^+^ IL-17^+^ CD4^+^ T-cell responses were detected (data not shown). To exclude that this was not due to the stimulation method, we used KWC to stimulate the cytokine (IL-17/IL-10/IFN-γ) production by activated CD4^+^ T cells, nonspecific/non-proliferating CD4^+^ T cells and CD4^−^ cells (control). The cytokine concentrations in the supernatants confirmed that the SA-specific CD4^+^ T cells produced IL-17 and IFN-γ, but likely not IL-10 (though no positive control for IL-10 production was included), as shown in Figure 2f.

To assess the potential presence of SA-specific memory T cells, we cultured naïve (CD45RA^+^) and memory (CD45RA^−^) CD4^+^ T cells (obtained from two donors) with (CD14^+^) monocytes that were either pulsed with KWC or TRAP, or left unpulsed (Fig. S1). While responses of the initially naïve T cells were low, SA-specific memory T-cell responses were readily detectable (i.e., ≤0.9% and ≤18.5% of proliferating CD4^+^ T cells, respectively).

### Phenotypic plasticity of SA-specific T-cells

The functional properties of Th17 cells can be affected under polarizing conditions induced by infection or immunization.^25^ Using the Th1/Th17-inducing antigens EbhA, IsaA and SdrE, we studied the plasticity of responding cells in terms of CD4^+^ T-cell proliferation and cytokine (IL-17/IFN-γ/IL-10) production, using *in vitro* polarization by modulatory pro- or anti-inflammatory cytokine combinations.

**Figure 3.**
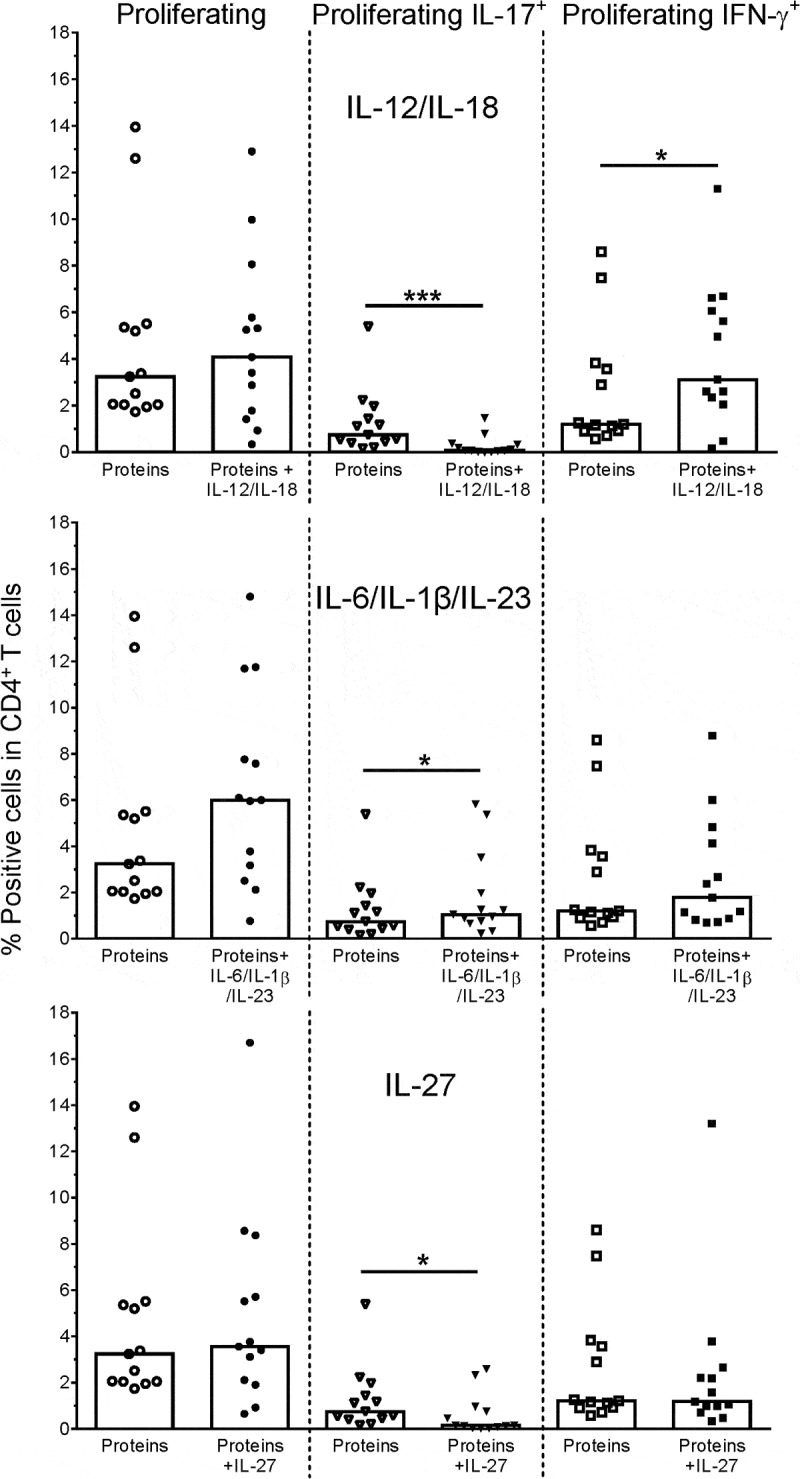
**Plasticity of SA-specific CD4^+^ T cells**. Proliferation and cytokine (IL-17, IFN-γ) production of antigen-specific CD4^+^ T cells was evaluated by intracellular cytokine staining (ICS). Healthy donor PBMCs were stimulated with a pool of the Th17-driving SA proteins EbhA, IsaA, SdrE (‘Proteins’), and frequencies of responding cells in the CD4^+^ T cells were assessed in the absence or presence of modulating cytokines, i.e., IL-12/IL-18 or IL-6/IL-1β/IL-23 combinations, or IL-27. Bars represent medians. Each symbol represents one individual. * *P*< .05; *** *P*< .001. Cytokine concentrations in culture supernatants assessed using cytometric bead array were found to be consistent with the ICS data (data not shown).

In the presence of the Th1-polarizing cytokines IL-12 and IL-18,^37,38^ proliferation was not significantly affected, while IL-17 production was significantly reduced and IFN-γ production tended to be slightly increased (*p* = .0002 and *p* = .03, respectively; Figure 3). The presence of the Th17-inducing cytokines IL-6, IL-1β and IL-23^26,39,40^ did not significantly stimulate proliferation or IFN-γ production, and appeared to increase IL-17 production (*p* = .01), suggesting that the responding Th17 cells were almost fully differentiated. In the presence of the anti-inflammatory cytokine IL-27, neither the proliferation nor the IFN-γ production was significantly affected, but IL-17 production tended to be decreased (*p* = .02).

No IL-10 expression by the responding cells was detected, under any of the experimental conditions (data not shown). This was expected for IL-6, IL-23 and IL-1β,^41^ but not for IL-27 which is known to stimulate IL-10 production by CD4^+^ T cells.^42^ Considering the used experimental protocol, this result does not, however, exclude the presence of early IL-10 responses by innate immune cells, the levels of which may have been substantially reduced after 6–7 days by cytokine consumption and/or degradation.

The cytokine concentrations in the associated culture supernatants were aligned with the ICS data (data not shown).

### Identification of IL-10-producing cells

**Figure 4.**
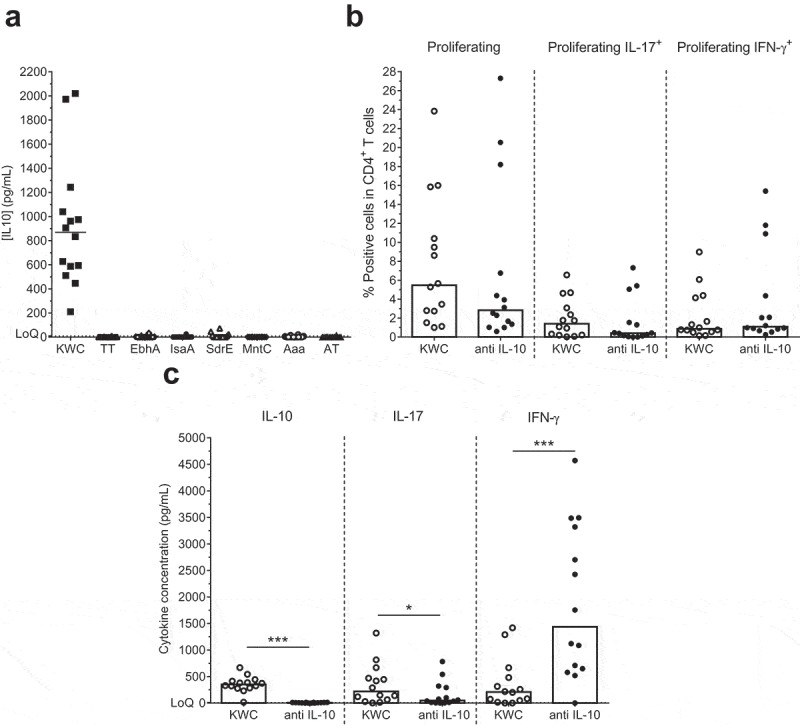
**IL-10 production by SA-activated cells**. (**a**) Background (medium) subtracted IL-10 concentrations in supernatants of PBMCs that were cultured overnight with either inactivated SA (killed whole cell antigen; KWC), tetanus toxoid (TT; control) or individual SA antigens, are shown. LoQ: Limit of Quantification. (**b**) Proliferation and cytokine (IFN-γ, IL-17, IL-10) production of KWC-stimulated cells in the absence or presence of anti-IL-10 antibody are represented. (**c**) Shown are the background (medium)-subtracted median cytokine (IL-10, IL-17, IFN-γ) concentrations measured in the supernatants of the cells presented in (**b**). * *P*< .05; *** *P*< .001. Each symbol in (**a-c**) represents one individual.

Having established that the elicited SA-specific Th1/Th17 cells did not produce IL-10, we further assessed the potential presence of pathogen-induced IL-10 responses. Stimulation with KWC, but not with any of the six antigens or negative control, increased the IL-10 concentrations in the culture supernatants (Figure 4a). The early time-point of the IL-10 detection (i.e., a few hours post stimulation; data not shown) suggested an origin from innate immune cells. Since CD141^+^ HLA-DR^+^ dermal dendritic cells (dermal DCs) can reside in perivascular locations in the upper dermis, and have a tolerogenic activity that is mediated via IL-10 expression,^43^ further characterization of the KWC-activated IL-10-producing cells was performed by staining for expression of CD141 (a dermal DC marker^43^), CD1a (a conventional DC marker), CD14 (a marker for monocytes/macrophages) and HLA-DR. The data revealed that the cells exhibited a CD14^+^ HLA-DR^+^ CD141^+^ CD1a^−^ phenotype (Fig. S2).

We then studied the effect of IL-10 on responding Th1 and Th17 cells, by evaluating the IL-17 and IFN-γ production by KWC-stimulated CD4^+^ T cells in the presence or absence of anti-IL-10 antibody. After addition of the antibody, no statistically significant effects were observed on the IFN-γ expression, proliferation or the IL-17 expression (Figure 4b), though the latter two functions tended to be slightly decreased. In the corresponding supernatants, addition of the antibody tended to result in decreased concentrations of both IL-10 (the control) and IL-17 (*p* = .0001 and *p* = .01, respectively), while IFN-γ concentrations were increased (*p* = .0005; Figure 4c). Though there might have been a minor impact on cell proliferation, this suggests that the changes in cytokine concentrations were more likely functional than frequency-induced. Overall, the data suggest that the IL-10 blockade mainly affected Th1 responses, by increasing IFN-γ production and reducing IL-17 production, and thus shifting the Th1/Th17 balance toward Th1. This confirmed the above-mentioned effects of IL-12 and IL-18 on the Th17 responses.

### Impact of vaccination on SA-specific memory T cells

**Figure 5.**
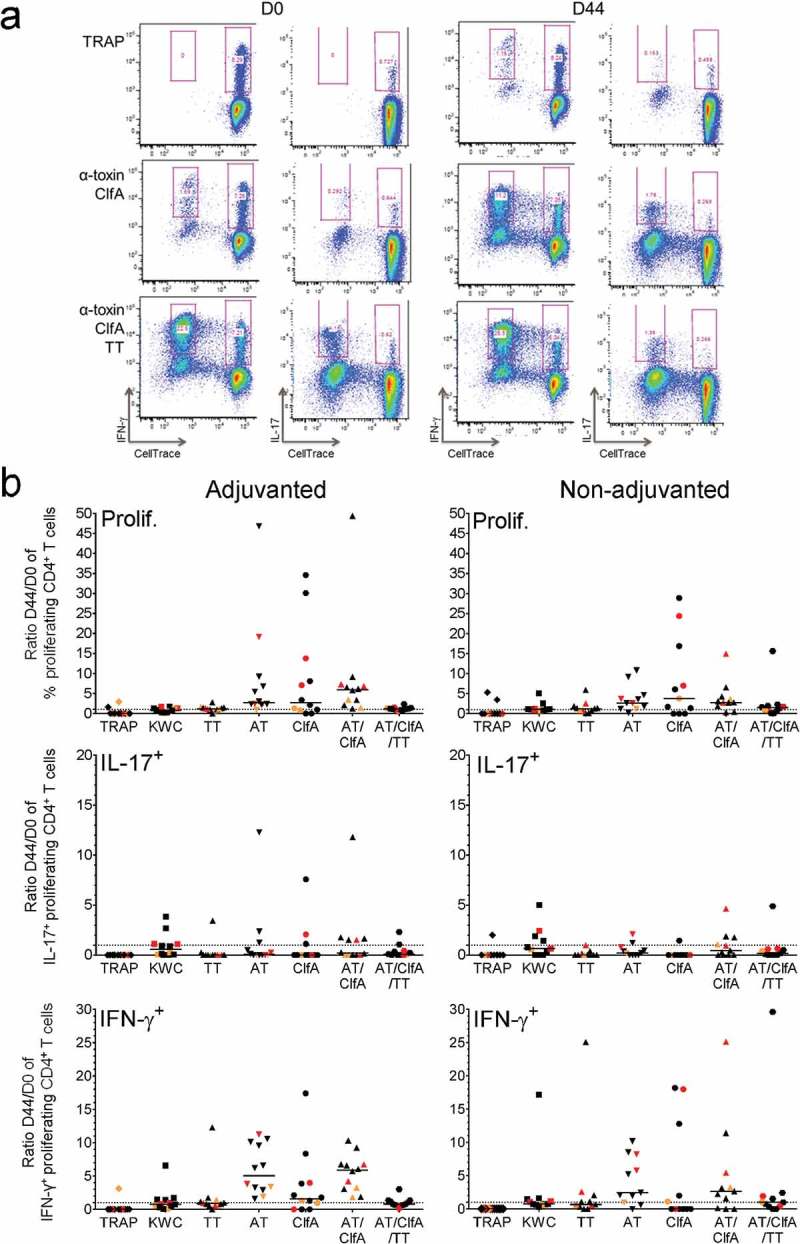
**Impact of vaccination on SA-specific memory CD4^+^ T cells**. Proliferation and cytokine production by CD4^+^ T cells was determined using intracellular cytokine staining and flow cytometry upon 7 days of *in vitro* stimulation of PBMCs derived from blood samples from vaccinees. Subjects received AS03-adjuvanted or non-adjuvanted multicomponent (CP5/CP8/AT/ClfA) SA candidate vaccine. (**a**) The gating strategy to identify the specific proliferating (‘CellTrace’-labeled) cytokine-producing CD4^+^ T cells at pre-vaccination (D0) and two weeks post-dose 2 (D44) in a blood sample from a recipient of AS03-adjuvanted vaccine is presented. Gating was performed on CD4^+^CellTrace^low^ cells. The numbers in pink font represent the percentage of each distinct population based on its cytokine (IFN-γ, IL-17) production. The presented results are representative of the range of responses seen with all subjects in the analysis. (**b**) D44/D0 ratios between the background (medium)-subtracted frequencies of all proliferating CD4^+^ T cells and of IL-17^+^ and IFN-γ^+^ proliferating CD4^+^ T cells are represented for recipients of AS03-adjuvanted or non-adjuvanted vaccine (N = 11 and N = 12, respectively). Each symbol represents one individual, with colors indicating their carrier status at baseline or post-vaccination (non-carrier: black; intermediate carrier: orange; carrier: red). Overall, distributions of non-carriers/intermediate carriers/carriers at baseline were 73/9/18% and 75/17/8% in the non-adjuvanted and AS03-adjuvanted groups, respectively. The distributions of non-carriers/carriers at D30 were 82/11% and 83/17% in the non-adjuvanted and AS03-adjuvanted groups, respectively. Solid lines represent medians. Dotted lines represent the limit of quantitation. KWC, inactivated SA ‘killed whole cell’ control antigen. TT, tetanus toxoid (positive control). Prolif, proliferation.

Next, we evaluated the impact of vaccination on the proliferation and IL-17 and IFN-γ production by pre-existing SA-specific memory T cells. From a trial evaluating SA candidate vaccines containing CPS5/8 conjugated to TT, AT and ClfA,^30^ we used blood samples collected before vaccination (D0) and two weeks after the second dose (D44) from healthy subjects who received either AS03-adjuvanted^44^ (N = 12) or non-adjuvanted (N = 11) vaccine. The participants were on average 31 years of age (range 21 to 40) and predominantly male (52%) and non-carrier (72–83%, depending on the vaccine group and time-point; see Figure 5).

Memory CD4^+^ T-cell responses were evaluated after long-term stimulation with AT and ClfA (Figure 5a). In these experiments, AT and ClfA were used either separately, or in combination both without and with TT (the latter was done to allow comparison with the TT-specific clinical trial data). In terms of proliferation, stimulation with either antigen elicited low-magnitude antigen-specific recall responses in the majority of subjects of both the AS03-adjuvanted and non-adjuvanted groups (median D44/D0 ratios: ≤6.0% and ≤3.8%, respectively; Figure 5b). The vaccination also induced moderate expansion of pre-existing antigen-specific IFN-γ^+^ cells (median ratios ≤5.9% and ≤2.6% in the AS03- and non-adjuvanted groups, respectively), but not of any pre-existing IL-17^+^ cells, which were only observed in 1 to 5 participants per group (at median ratios <1%). Regardless of the presence of adjuvant, the response ratios tended to be lower when the stimulation with the two SA antigens (combined or individually) was performed in the presence of TT, *versus* without TT. No clear trend associated with the subjects’ carriage statuses was observed, though the low number of samples per category did not allow drawing definitive conclusions based on the carrier status. There was also no evidence of statistically significant differences between the corresponding AS03-adjuvanted and non-adjuvanted groups, for any of the antigens.

## Discussion

Pathogen-induced IL-10 mediated downregulation and modulation of vaccine-induced T-cell responses constitute a challenge for the development of certain anti-bacterial vaccines. Further insight into the nature of anti-bacterial T-cell lineages and phenotypic plasticity, either in steady state or under inflammatory conditions such as immunization, is therefore essential. Focusing on SA, our data show that (i) six out of the nine proteins evaluated acted as human Th1-driving antigens, and three of these proteins also elicited Th1/Th17 and/or Th17 responses, but none induced Th22 responses or IL-10 production by activated cells; (ii) IL-10, likely produced by innate immune cells, influenced mainly Th1 cells, by suppressing IFN-γ production; (iii) SA-specific Th17 cells retain a certain degree of plasticity; and (iv) consistent with the Phase I trial data, vaccination with AT and ClfA induced expansion of Th1 responses (which were most likely recruited from a pre-existing memory pool), but not of Th17 responses, and no significant adjuvant effect was observed.

Whether an efficacious vaccine requires a multi-antigen approach, or a single antigen promoting protective antibody and Th1/Th17 responses, is subject to debate.^21,23,45,46^ Here, the identified human CD4^+^ T-cell antigens differed in terms of the cellular phenotypes they induced. We identified effective activators of Th1 responses (EbhA, IsaA, SdrE, MntC, Aaa and AT), Th17 responses (EbhA, IsaA, SdrE, and AT) and Th1/Th17 responses (EbhA, IsaA, and SdrE). These results add to the previously reported human SA T-cell antigens, which include GlpQ, Plc, Geh, Lip, SplC and SplD,^9^ as well as the well-studied AT antigen, which was also previously found to induce Th1 and Th17 responses in humans.^47,48^ We identified MRPII, IsdA, and ClfA as less effective human T-cell antigens. Correspondingly, ClfA was previously also shown to induce only low-level Th1 responses and no Th17 responses in humans.^7,30^ We also found that none of the proteins induced an IL-22 response. IL-22 has been reported to play a role in controlling nasal colonization.^49^

SA can induce expression of the Th17-polarizing cytokines IL-6 and IL-23.^27^ Our data suggest that the Th1/Th17 cells responding to EbhA, IsaA and SdrE retained a certain degree of plasticity, in line with previous findings (reviewed in ref.^50^). Indeed, in the presence of IL-12 and IL-18 (which can synergistically induce IFN-γ production^37,38^), IFN-γ production increased, while IL-17 production decreased. Reduced IL-17 production was also observed in IL-27 supplemented cultures. We hypothesize that different mechanisms of IL-17 downregulation are at play, which are driven by the Th1/Th17 balance^51,52^ and/or by IL-10 production.^42,53^

The importance of the IL-17/IL-10 balance is highlighted by several studies. First, control of IL-10-induced immune suppression is critical, both for reducing the severity of SA bacteremia^29,54^ and for preserving vaccine-induced immunity. Indeed, SA can promote IL-10-dependent suppression of the production of cytokines (e.g., IL-2, IFN-γ or IL-1β) by innate cells and T cells, and inhibit antigen presentation by downregulating major histocompatibility complex (MHC) class II/HLA-DR and CD86 expression, thus dampening both the innate and adaptive immune responses.^27^ Second, the post-hoc analysis of the Phase IIb/III study of the V710 vaccine candidate, containing non-adjuvanted IsdB, demonstrated that undetectable serum IL-2 levels before vaccination and undetectable pre-operative IL-17 levels were correlated with postoperative mortality.^55^ These clinical data further stress the need for a better understanding of human immune responses to SA.

We did not observe IL-10^+^ CD4^+^ T cells after overnight or long-term (7 days) primary stimulation either with KWC or with any of the evaluated proteins, including those driving Th17 responses. This contrasts with reports showing that naive T cells can differentiate into IL-17^+^ IL-10^+^ CD4^+^ T cells after a 5-day stimulation with KWC.^26^ In our experiments, IL-10 responses in supernatants were rapid (within hours), and low relative to those in the reference study.^26^ Since both Th17 and innate cells can produce IL-10 upon *in vitro* stimulation,^26–28^ our results suggest that the observed IL-10 responses originated from innate cells rather than from CD4^+^ T cells. Interestingly, the phenotype of the IL-10-producing cells (CD14^+^ HLA-DR^+^ CD141^+^ CD1a^−^) resembled that of migratory tolerogenic dermal innate cells (monocytes and DCs). Such cells can produce IL-10, and have been detected in perivascular locations in the dermis, and (for CD141^+^ DCs) also in peripheral blood (albeit in low frequencies).^43^ However, this would need confirmation with respect to the potential expression of additional markers (CD83, CD86, CD80 and/or PD-L1), and the bacterial carriage statuses of the corresponding donors. Nevertheless, our experiments with anti-IL-10 antibody suggested a pathogen-induced immunosuppressive mechanism, whereby IL-10 (likely secreted by innate immune cells upon KWC stimulation) mainly suppressed the Th1 responses, resulting in enhanced Th17 responses. This could shift the Th1/Th17 balance toward a Th17-biased phenotype. The benefit of such a response in humans has been discussed above (see also refs.^54,55^).

In the Phase I trial, the candidate vaccines were shown to induce robust antibody responses against CPS5, CPS8, AT and ClfA, but only low-magnitude (<0.02%) AT and ClfA-specific Th1 responses, and no Th17 responses, irrespective of the presence of AS03.^30^ The current data, characterizing the AT- and ClfA-specific CD4^+^ T-cell responses that were induced either by the vaccination, or by *in vitro* stimulation using cells from non-vaccinees, were overall consistent with the trial data,^30^ although in our experiments, *in vitro* stimulation with ClfA induced neither proliferation nor IFN-γ production.

Compared to the trial data, the D44/D0 ratios of IFN-γ^+^ CD4^+^ T-cell responses observed here were ≤4-fold and ≤2-fold higher for AT and ClfA, respectively, suggesting that the prolonged stimulation protocol used here (7 days), and the similar protocols used in other studies^26^ were more effective in detecting Th1 responses than the short-term (20 h) stimulation used in the trial. This may be an SA-specific feature, since more robust IFN-γ^+^ CD4^+^ T-cell responses were elicited by AS03-adjuvanted influenza, HBV, or *Streptococcus pneumoniae* vaccines, using similar short-term stimulation protocols.^56–58^ The low detection of vaccine-induced Th17 responses in our experiments cannot be linked to the stimulation protocol; however, since Th17 responses typically require a time-frame of ≤5 days of stimulation.^39,59^ Thus, our data suggest that both AT and ClfA are ineffective activators of Th17 responses in the vaccine context.

Several limitations are noted in interpreting the current data. First, the lack of a clear impact of the subjects’ carriage statuses on the immune responses observed here and in the Phase 1 study^30^ may be due to the confounding effect of inter-subject variation. Such variation may have been present in the levels of priming immunity and immunomodulation exhibited by the carriers (as seen elsewhere^60^) as well as in the relative contributions of AT- and ClfA-specific memory responses, and is likely associated with the small sample sizes evaluated in both studies. Second, inferences from our study may also be limited by the lack of functional (neutralization or opsonization) data for the current set of SA proteins. While functional assays were performed for the vaccine-induced AT- and ClfA-specific responses in the Phase 1 trial,^30^ association of this data with the cell-mediated immune responses observed in the current study was not pursued here, due to the sample size limitations of the datasets for these specific proteins. Finally, the limitations of the current murine infection models emphasize the need to generate human immunology data in the context of clinical studies. Generating such data could guide the extrapolation of data from murine models.

### Conclusion

The Th1/Th17-driving antigens EbhA, IsaA and SdrE shared low IL-10-promoting abilities and restricted phenotypic plasticity under pro- and anti-inflammatory conditions. The current data may serve to extend the present knowledge of SA CD4^+^ T-cell antigens and Th17 immunology. Further analysis of the association between CD4^+^ T cell response profiles and susceptibility to infection may lead to a better understanding of protective immune response, and to the optimal antigen selection. However, given the multitude of SA virulence factors and its complex immunopathology, several hurdles remain to be surmounted for successful vaccine development. The availability of optimized clinical T-cell read-outs, able to differentiate between naturally protected and non-protected individuals, would further advance clinical vaccine evaluations.

## Methods

### Similarity analyses and antigen selection

Full genomes from seven SA strains (NC_002745.1/N315, NC_002758.1/Mu50, NC_003923.1/MW2, NC_002951.1/COL, NC_002745.1/NCTC8325, NC_002952.1/MRSA252, and NC_002953.1/MSSA476; National Center for Biotechnology Information [NCBI] GenBank) were screened for the presence of candidate vaccine antigens using NCBI BLAST software. Candidates were selected based on published data on cell surface-expressed protein features and on functional domains,^31–36^ as well as on the in-house assessment of intra-species conservation (see Table 1). The proteins were produced in *Escherichia coli* and purified by Hyglos GmbH (Bernried, Germany) using affinity chromatography with repeated column purification steps (EndoTrap red 5/1; #321063; final endotoxin concentrations were <0.5 EU/mL for all proteins except SdrE, which was 0.67 EU/mL).

### Human PBMCs

Written informed consent was obtained from the participants prior to the collection of all blood samples used in this study.

In the experiments assessing the impact of vaccination, cryopreserved PBMCs were derived from blood samples from a clinical study (NCT01160172^30^) evaluating AS03_B_-adjuvanted and non-adjuvanted SA candidate vaccines that contained TT-conjugated CPS5 and CPS8 (10 µg each per dose), and detoxified AT H35R mutant and ClfA mutant (both at 30 µg per dose). AS03_B_ (elsewhere in this article referred to as AS03) is an Adjuvant System containing α-tocopherol and squalene in an oil-in-water emulsion (5.93 mg tocopherol).^44^ The trial was approved by the Centre Hospitalier Universitaire de Tivoli (La Louvière, Belgium) Ethic Committee, and conducted in accordance with the Helsinki Declaration.

The blood samples used in the current research work were collected from 23 recipients of adjuvanted or non-adjuvanted vaccines (N = 12 and N = 11, respectively). Samples were obtained at D0, and at D44 (generally at the peak of CD4^+^ T-cell responses). Individual and group median D44/D0 CD4^+^ T-cell response ratios were calculated. In the trial, subjects were characterized at baseline, as either non-carriers (SA-negative at screening and D0), intermediate carriers (SA detected either at screening or D0), or carriers (SA-positive in ≥1 sample at screening and D0), and at D30, as either non-carriers (SA-negative), or carriers (SA-positive in ≥1 sample). In all other experiments, cryopreserved PBMCs were derived from 20 healthy donor blood samples (N = 14 to 16 per experiment) derived from the Établissement de Transfusion Sanguine (ETS), Charleroi, Belgium. The Hospital Erasme (Brussels, Belgium) Ethic Committee was notified of the use of PBMCs from ETS for the current research work.

### CD4^+^ T cells and cytokines

SA-specific CD4^+^ T-cell responses were assessed *in vitro* using standard protein antigen-stimulated PBMC read-outs. Briefly, PBMC were thawed, labeled by fluorescent proliferation dye (CellTrace Pacific Blue #C344557; Thermo Fisher), and stimulated *in vitro* with the SA proteins, or with KWC (positive control; 5 µg/mL). SA proteins were used either individually (see Figures (1, 2, 4a and 5)), or pooled (see Figures 3 and 5: EbhA/IsaA/IsdrE [10 µg/mL each] were used in ratio 1:1:1; and AT/ClfA [10 µg/mL each] were used without or with the positive control TT [at 5 µg/mL] in 1:1 or 1:1:1 ratios, respectively). KWC was composed of a pool of four SA strains, each cultured in the optimal condition for expression of CPS8 and CPS5, i.e., liquid (NRS482 and Wright brain-heart infusion) and gel (Wright 6% NaCl/Lowenstein solutions), respectively. TRAP (GSK) was the negative control.

In experiments evaluating T-cell plasticity (see Figure 3), the culture medium was supplemented with modulatory cytokines (i.e., IL-12/IL-18, both at 0.5 ng/mL; IL-27, at 200 ng/mL; or IL-6/IL-1β/IL-23, each at 50 ng/mL). In experiments assessing the influence of IL-10 (see Figure 4(b and c)), the culture medium was supplemented with anti-IL-10 antibody (2 µg/mL).

After 6 or 7 days of culture, the culture supernatants were collected for measurement of cytokine (IFN-γ/TNF-α/IL-17/IL-22/IL-10/IL-1β/IL-6) concentrations using CBA kits (BD Biosciences; Figures 1b and 4c), or the CD4^+^ T-cell proliferation was measured using fluorescence-activated cell sorting (FACS; see Figure 1a). Alternatively, after the 6–7 days of culture, the labeled PBMCs were stimulated non-specifically for 3–4 h with *p*-phorbol-12-myristate-13-acetate (100 ng/mL) and ionomycin (1 µg/mL; both Sigma-Aldrich) at 37°C, in the presence of GolgiPlug-containing brefeldin A (BD Biosciences). Subsequently, CD4^+^ T-cell proliferation was measured by fluorescence-activated cell sorting (FACS), and/or the cells were permeabilized, stained and analyzed for cytokine (IFN-γ/IL-17/IL-10/IL-22) production, using ICS and flow cytometry as described previously^61^ (see Figures 2(a–e), 3, 4b and 5).

### Identification of IL-10-expressing cells

The presence of IL-17^+^ IL-10^+^ CD4^+^ T cells was assessed in three experiments, using PBMCs derived from six donors in total. After long-term stimulation with KWC as described above, cells were harvested and FACS-sorted into populations of proliferating SA-specific CD4^+^ T cells (CD4^+^ CellTrace^−^), non-proliferating CD4^+^ T cells (CD4^+^ CellTrace^+^) and non-proliferating non-CD4^+^ cells (CD4^−^ CellTrace^+^). Sorted cells were stimulated non-specifically (as described above), and cytokine (IL-17/IFN-γ/IL-10) concentrations in supernatants were measured by CBA (see Figure 2f).

In other experiments, non-labeled PBMCs were stimulated overnight with the SA antigens. Then, either the IL-10 concentrations were measured in supernatants (Figure 4a), or the IL-10 producing cells were analyzed for CD141, HLA-DR, CD1a and/or CD14 expression, using flow cytometry (Fig. S2).

### Memory CD4^+^ T cells

SA-specific memory responses were assessed by isolating (CD3^+^) CD4^+^ CD45RA^+^ naïve T cells, (CD3^+^) CD4^+^ CD45RA^−^ memory T cells and CD14^+^ monocytes, by FACS. Proliferation was monitored by culturing CellTrace-labelled naïve and memory CD4^+^ T cells with monocytes that were either pulsed overnight with KWC or TRAP, or left non-pulsed (ratio CD4^+^ T cells: monocytes 2:1), for 7 and 14 days to detect memory and naïve responses, respectively (see Fig. S1).

### Statistical analyses

Statistical significance was determined on background (medium)-subtracted responses using the Wilcoxon matched-pairs signed rank test or the Mann Whitney test without correction for multiplicity, using GraphPad Prism version 6.02 (GraphPad Software, San Diego, USA).
