# Supplementary material for: Role and plasticity of Th1 and Th17 responses in immunity to Staphylococcus aureus
Source: Hum Vaccin Immunother. 2019 Oct 31;15(12):2980–92. doi: 10.1080/21645515.2019.1613126 (PMC6930085; doi:10.1080/21645515.2019.1613126)
Supplement: Supplemental Material [file khvi-15-12-1613126-s001.zip › FigS1.pptx]

## Slide 1
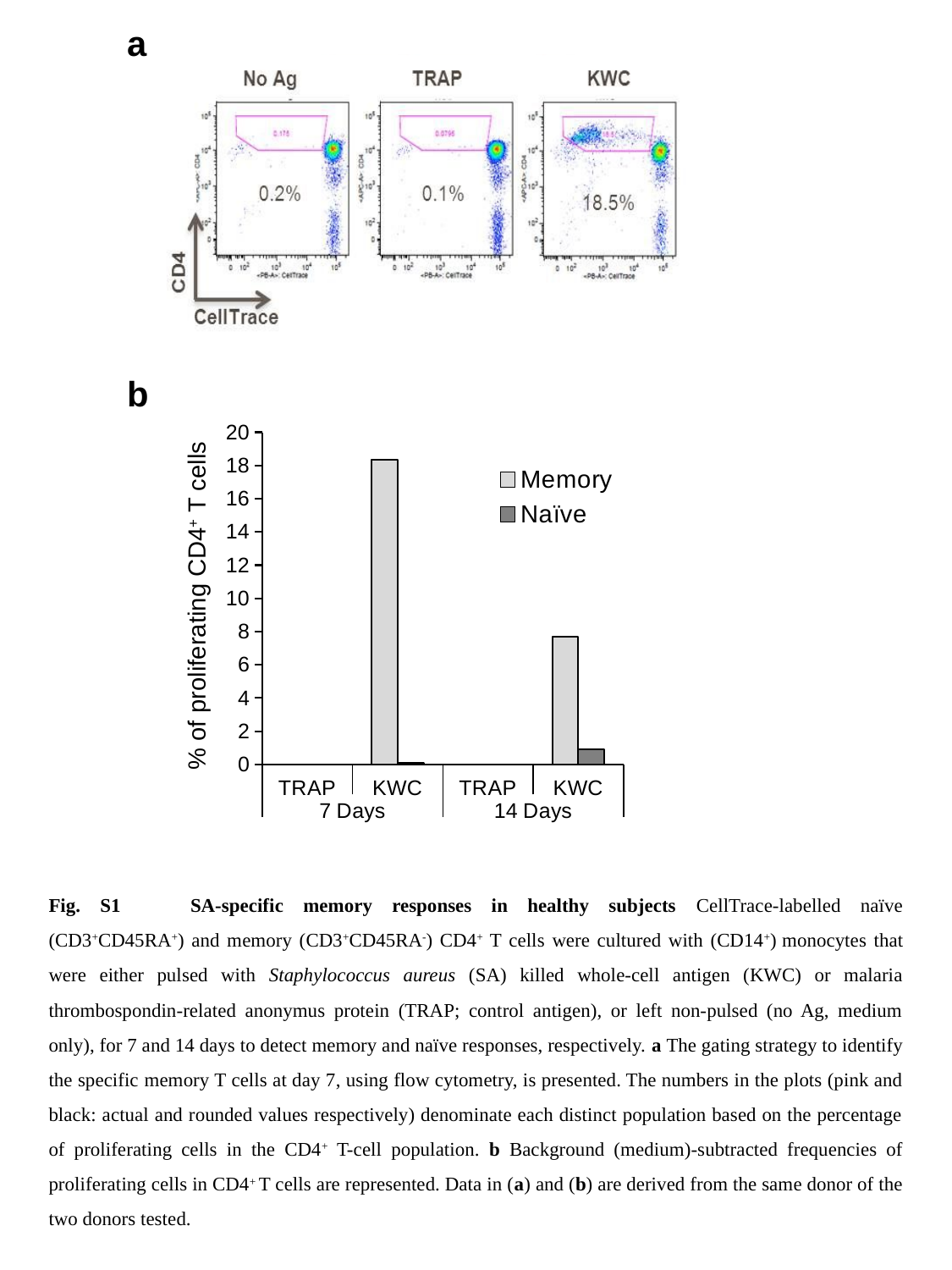

a
b
### Chart
| Category | Memory | Naïve |
|---|---|---|
| TRAP | 0.0 | 0.0 |
| KWC | 18.33 | 0.08000000000000002 |
| TRAP | 0.0 | 0.0 |
| KWC | 7.7 | 0.9000000000000001 |% of proliferating CD4+ T cells
Fig. S1	SA-specific memory responses in healthy subjects CellTrace-labelled naïve (CD3+CD45RA+) and memory (CD3+CD45RA-) CD4+ T cells were cultured with (CD14+) monocytes that were either pulsed with Staphylococcus aureus (SA) killed whole-cell antigen (KWC) or malaria thrombospondin-related anonymus protein (TRAP; control antigen), or left non-pulsed (no Ag, medium only), for 7 and 14 days to detect memory and naïve responses, respectively. a The gating strategy to identify the specific memory T cells at day 7, using flow cytometry, is presented. The numbers in the plots (pink and black: actual and rounded values respectively) denominate each distinct population based on the percentage of proliferating cells in the CD4+ T-cell population. b Background (medium)-subtracted frequencies of proliferating cells in CD4+ T cells are represented. Data in (a) and (b) are derived from the same donor of the two donors tested.
