# Supplementary material for: Role and plasticity of Th1 and Th17 responses in immunity to Staphylococcus aureus
Source: Hum Vaccin Immunother. 2019 Oct 31;15(12):2980–92. doi: 10.1080/21645515.2019.1613126 (PMC6930085; doi:10.1080/21645515.2019.1613126)
Supplement: Supplemental Material [file khvi-15-12-1613126-s001.zip › FigS2_revised.pptx]

## Slide 1
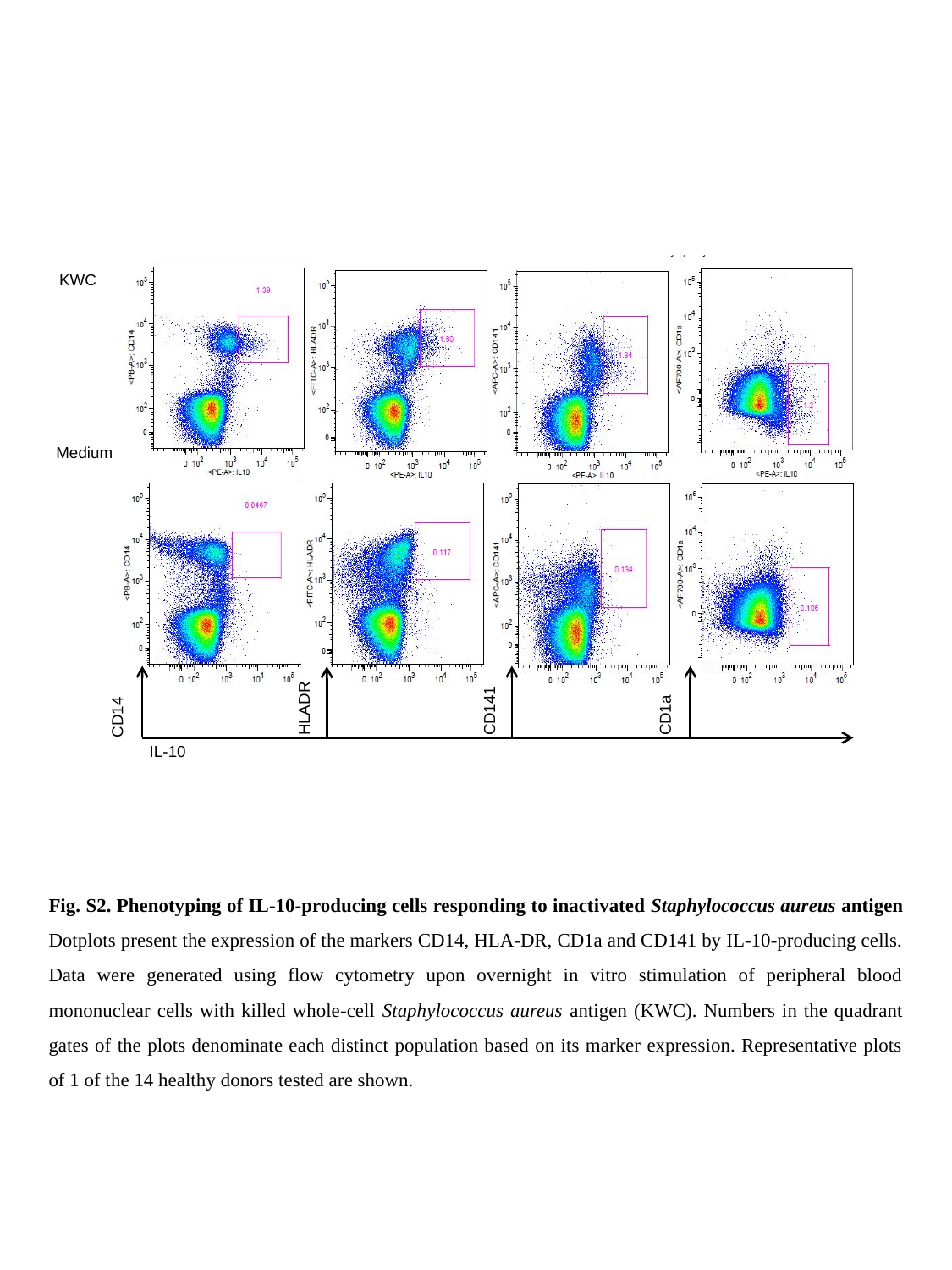

KWC
Medium
HLADR
CD141
CD1a
CD14
IL-10
Fig. S2. Phenotyping of IL-10-producing cells responding to inactivated Staphylococcus aureus antigen Dotplots present the expression of the markers CD14, HLA-DR, CD1a and CD141 by IL-10-producing cells. Data were generated using flow cytometry upon overnight in vitro stimulation of peripheral blood mononuclear cells with killed whole-cell Staphylococcus aureus antigen (KWC). Numbers in the quadrant gates of the plots denominate each distinct population based on its marker expression. Representative plots of 1 of the 14 healthy donors tested are shown.
